# Mitochondrial Gene Expression and Beyond—Novel Aspects of Cellular Physiology

**DOI:** 10.3390/cells9010017

**Published:** 2019-12-19

**Authors:** Anna V. Kotrys, Roman J. Szczesny

**Affiliations:** Institute of Biochemistry and Biophysics, Polish Academy of Sciences, 02-106 Warsaw, Poland; akotrys@ibb.waw.pl

**Keywords:** mitochondria, mitochondrial gene expression, mtDNA, mtDNA transcription, mtRNA, post-transcriptional mtRNA processing, dsRNA, innate immunity, interferon response

## Abstract

Mitochondria are peculiar organelles whose proper function depends on the crosstalk between two genomes, mitochondrial and nuclear. The human mitochondrial genome (mtDNA) encodes only 13 proteins; nevertheless, its proper expression is essential for cellular homeostasis, as mtDNA-encoded proteins are constituents of mitochondrial respiratory complexes. In addition, mtDNA expression results in the production of RNA molecules, which influence cell physiology once released from the mitochondria into the cytoplasm. As a result, dysfunctions of mtDNA expression may lead to pathologies in humans. Here, we review the mechanisms of mitochondrial gene expression with a focus on recent findings in the field. We summarize the complex turnover of mitochondrial transcripts and present an increasing body of evidence indicating new functions of mitochondrial transcripts. We discuss mitochondrial gene regulation in different cellular contexts, focusing on stress conditions. Finally, we highlight the importance of emerging aspects of mitochondrial gene regulation in human health and disease.

## 1. Introduction: The Role of Mitochondria within the Cell

Mitochondria are present in the majority of eukaryotic cells, where they play a central role in many processes. They are hubs of energy production as sites of oxidative phosphorylation [1]. They are also engaged in maintaining an appropriate redox state and recycling oxidized electron carriers that are important for cell proliferation [2,3]. Moreover, mitochondria play a key role in cellular signaling, buffering calcium ions and regulating apoptosis processes [4,5]. Mitochondria are also the source of most of the cellular reactive oxygen species (ROS), which may impact on various cellular process [6,7]. Mitochondria take part in response to external stimuli, e.g., viral infection [4], and they are also the place where many basic processes related to innate immune intersect [8,9]. Mitochondrial malfunction is related to numerous pathological states in humans, such as cancer and neurodegeneration [10,11,12]. Furthermore, many human hereditary diseases are caused by mitochondrial dysfunction. Mutations in the mitochondrial genome or nuclear genes that encode mitochondrial proteins lead to primary and secondary mitochondrial diseases connected with improper mitochondrial function [13,14]. In this review, we will focus on the emerging aspects of mitochondrial biology and its implications in human health and we guide interested readers to detailed reviews on mitochondrial disorders [15,16,17].

Mitochondrial biogenesis is far more complex in comparison to other cellular organelles since they possess their own genome, which requires dedicated gene expression machinery [18,19,20]. As a consequence, mitochondrial protein production must be coordinated with the nucleocytoplasmic compartment for proper organelle homeostasis. Many nucleus-encoded proteins are needed for mitochondrial transcription, RNA processing, degradation and translation; all to produce just over a dozen proteins that are parts of the oxidative phosphorylation complexes encoded by the mitochondrial genome [18,19]. Recently, new findings deciphering the link between mitochondrial gene expression and cellular homeostasis were reported. Here, we review the basis of mitochondrial gene regulation with a focus on recent findings, highlighting new RNA-driven mechanisms by which mitochondria contribute to the regulation of cell biology in human health and disease.

## 2. Mitochondrial Genome—A Simple Molecule with Unorthodox Organization

**Figure 1:**
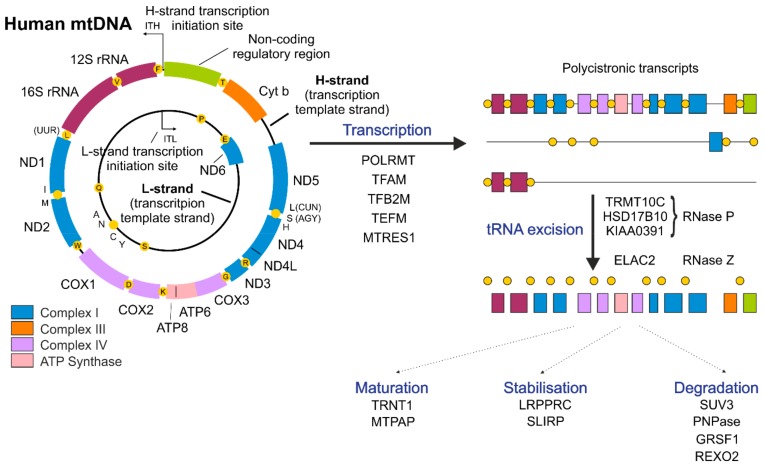
Schematic of human mtDNA and basic steps of mtRNA metabolism. Human mtDNA is a circular double-stranded molecule. Marked are template heavy (H-strand) and light (L-strand) strands of mtDNA. Marked are genes encoding subunits of Complex I (blue), III (orange), IV (violet) and subunits of ATP Synthase (pink). ND4/ND4L and ATP6/ATP8 open reading frames are overlapping and are included in bicistronic mRNAs. Genes encoding rRNAs are colored purple. Genes encoding tRNAs are represented as yellow dots with single letter code depicting their aminoacids. Mitochondrial transcription is initiated from L- and H- strand promoters (ITL and ITH, respectively) located within non-coding regulatory region (NCR). Transcription is driven by DNA-dependent RNA polymerase (POLRMT) with help of its co-factors: TFAM, TFB2M, TEFM and MTRES1. Mitochondrial transcription leads to the formation of three polycistronic transcripts that undergo further processing. Most of mitochondrial mRNAs and rRNAs are punctuated by tRNAs which are excised from the nascent RNA precursors by RNAse P and Z. Arisen transcripts may be subjected to various subsequent processes as maturation, stabilization, degradation, and modification. Selected factors engaged in these processes are presented.

Mitochondria are semiautonomous organelles possessing their own genome. Human mitochondrial DNA (mtDNA) is a ~16 kb circular molecule composed of double-stranded DNA [21]. Human mtDNA contains only a few genes, but all of them are essential for proper cell function, and mutations in mtDNA have dire consequences [13]. Although human mitochondria contain more than 1100 proteins [22], only 13 of them are encoded by mtDNA. The remaining 24 of the 37 genes present in human mtDNA encode RNAs required for the mitochondrial translational apparatus (22 tRNAs and 2 rRNAs) [19] (Figure 1). The rest of the proteins necessary for mitochondrial function are encoded in the nuclear genome and are imported into mitochondria upon synthesis in the cytoplasm [23]. These proteins are key factors in mtDNA replication, transcription and translation processes [23,24]. The presence of a distinct mitochondrial genome is linked to the endosymbiotic origin of this organelle [25,26]. Recent studies of the mitochondrial genome indicated that mitochondria evolved from a proteobacterial lineage [27]. The human mitochondrial genome was first described in the early 1960s and was one of the first genomes to be fully sequenced [28,29]. Since then, the molecular mechanisms of mtDNA expression have been intensively investigated; nevertheless, our knowledge about these processes is still not complete.

The human mitochondrial genome is tightly packed in nucleoprotein complexes called nucleoids [30]. The application of super-resolution microscopy and mass spectrometry-based techniques significantly contributed to the determination of the nucleoid characteristics [31,32,33,34]. It was shown that each nucleoid contains one or several copies of mtDNA [33,34] and is spatially organized by DNA-binding proteins [31]. More than 20 proteins were identified to copurify with mitochondrial nucleoids [32], among which the most abundant protein mitochondrial transcription factor A (TFAM) that may serve as a mtDNA copy number regulator [35]. Discrete nucleoid complexes are dispersed among the mitochondrial network in the cell [33]. An estimated number of mtDNA molecules per mitochondrion may vary greatly between tissues and range from less than a hundred to thousands [36,37,38]. Each mtDNA molecule can be independently segregated in daughter cells, as nucleoids are exchanged within the mitochondrial network upon fusion/fission processes [39,40]. More than one mtDNA sequence can be present within an individual, leading to so-called mitochondrial heteroplasmy. This can be caused by somatic mutations, heteroplasmy of the oocyte or paternal leakage of mtDNA [41,42]. For a long time, mtDNA was considered to be strictly maternally inherited [43]; nevertheless, few reports suggest paternal contribution in the inheritance of the mitochondrial genome [44,45].

There are several models describing the mechanism of mtDNA replication. Notably, the proposed models are not mutually exclusive. Instead, they were proposed to operate in a complementary mode depending on the tissue, cellular state or energy demand [46,47,48]. For a detailed review of mtDNA replication, please see Holt and Reyes [49] and Gustafsson et al. [50].

Notably, mutations in nuclear-encoded mitochondrial proteins that participate in mtDNA maintenance are implicated in mitochondrial disorders [51]. To date, nearly 300 pathogenic point mutations have been reported in the DNA polymerase gamma (POLG) gene encoding mtDNA replicase, and POLG mutations are one of the main causes of inherited mitochondrial disorders [51,52].

## 3. Mitochondrial Transcription

MtDNA is composed of heavy (H-strand) and light (L-strand) strands (Figure 1) that can be distinguished by different sedimentation attributes in buoyant density ultracentrifugation due to the uneven distribution of guanines between DNA strands [21]. In humans, the G-rich H-strand serves as a template for the transcription of most mitochondrially encoded genes, while the transcription of the complementary L-strand results in the formation of mostly non-coding RNA (ncRNA) [53]. Only one protein-coding gene and 8 tRNAs are transcribed from the L-strand [53] (Figure 1). The exceptional ~1 kb, non-coding regulatory region (NCR) plays an important role both in mtDNA replication and transcription. The NCR plays an important role as a site of H-strand synthesis initiation [49,54]; moreover, transcription initiation start sites for both mtDNA strands (ITL, light-strand transcription initiation site, ITH, heavy-strand transcription initiation site) are also located within the NCR. The synthesis of RNA starts in both directions within the NCR and leads to the production of long, polycistronic transcripts (Figure 1) [55].

The human transcription apparatus appears to be simple and composed of a monomeric RNA polymerase (POLRMT) that is homologous to bacteriophage polymerases and only a few co-factors [19]. In addition to POLRMT, basic factors participating in the mitochondrial transcription process include TFAM, mitochondrial transcription factor B2 (TFB2M) and mitochondrial transcription elongation factor (TEFM) (Figure 1) [19,56]. Initial in vitro experiments showed that transcription can proceed with the presence of only two proteins: POLRMT and TFB2M. Nevertheless, the efficiency of this transcription apparatus is rather low [57]. Thanks to recent structural studies, the detailed step-by-step initiation of mitochondrial transcription was revealed [56]. It was shown that mitochondrial transcription is initiated by the binding of TFAM to the promoter region and the following recruitment of POLRMT to TFAM-bound mtDNA near the transcription start site [58,59]. Next, TFB2M is recruited to induce and stabilize the open conformation of mtDNA. After initiation of RNA synthesis, TFAM and TFB2M are subsequently released, and the elongation factor TEFM is recruited to enable the transition into transcription elongation [56]. TEFM was proposed to increase the processivity of POLRMT and to enable nearly whole-genome transcription [60,61,62]. Additionally, TEFM was shown to participate in the regulation of a replication/transcription switch [62,63,64]. Recently, TEFM was also proposed to play a role in mitochondrial RNA (mtRNA) processing [65].

Termination of mtDNA transcription is far less understood than its initiation. Mitochondrial transcription termination factor 1 (MTERF1) plays an important role in the termination of mtDNA transcription initiated from the ITL; however, it is not clear which factors take part in the termination of mtDNA transcription initiated from the ITH [56]. MTERF1 binds specific sequences within the tRNA^Leu^ gene, causing DNA unwinding and base flipping (i.e., rotation of the nucleotide base outside the DNA double helix), leading to transcription termination [66,67]. MTERF1 was also suggested to prevent interference of the transcription complexes operating in opposite directions [68] and to prevent collision of transcription and replication machineries [69]. Several other MTERF proteins are conserved in vertebrates and plants [70]. Despite playing a role in mitochondrial gene expression, none of the MTERF2-4 proteins were shown to act as a transcription terminator in mammals [71,72,73]. This raises the question whether there are other termination factors yet to be discovered in the human mitochondria.

Among mitochondrial transcription machinery constituents, only TFAM mutations are well established to contribute to human disease [17]. TFAM binds DNA in both sequence-specific and nonspecific manners. The former enables the initiation of mitochondrial transcription [58], and the latter enables the compaction of the mitochondrial genome [74,75]. As mtDNA transcription by POLRMT may serve as a source of RNA primers for mtDNA replication [76], it seems that both sequence-specific and nonspecific manners of TFAM DNA-binding contribute to the maintenance of the mtDNA copy number (reviewed in [77]). TFAM knockout in mice causes severe depletion of mtDNA and is embryonically lethal [78]. In humans, mutations in the TFAM gene were shown to cause a decreased mtDNA copy number and impaired cellular respiration underlying progressive liver failure with neonatal onset [79]. Altered TFAM levels and related changes in the mtDNA copy number were also proposed to be associated with neurodegeneration [80].

Recently, some novel factors participating in mitochondrial transcription regulation were reported. Among them, an interesting example pertains to mitochondrial transcription rescue factor 1 (MTRES1), which was shown to interact with POLRMT and TFAM and to prevent stress-induced loss of mtRNAs by acting at the mitochondrial transcription initiation level [81]. Another example refers to mitochondrial ribosomal protein L7/L12 (MRPL12), whose mutations may lead to respiratory chain deficiency that manifest as growth retardation and neurological deterioration [82]. In addition to being a constituent of the mitochondrial ribosome, MRPL12 also exists in a “free”, ribosome-unrelated matrix pool [83]. MRPL12 was shown to interact with POLRMT to regulate transcription [84]. It was proposed that MRPL12 may serve to coordinate transcription, ribosome biogenesis and/or protein synthesis processes [83,84]. Different roles of MRPL12 in mitochondrial gene regulation may be connected with the presence of two forms, the short and long forms, generated by proteolytic cleavage upon being imported into mitochondria, which may have distinct properties [85]. Several other factors, such as hormones, nuclear transcription factors and chromatin remodeling enzymes, were proposed to regulate mitochondrial transcription either by direct binding to mtDNA or by indirect regulation (reviewed in [86]).

## 4. Post-Transcriptional Regulation of mtRNAs

Mitochondrial transcription spans almost the entire mitochondrial genome, leading to the formation of three polycistronic transcripts (Figure 1) [19,55]. Two of them, resulting from transcription of either the H- or L-strand, encompass almost the entire genome and carry sequences corresponding to mRNAs, tRNAs, rRNAs and ncRNAs. The third transcript covers genes for two tRNAs (Phe and Val) and both rRNAs [19]. Further steps of cleavage and processing are required to obtain mature, functional RNAs [87].

In nascent precursor transcripts, most mitochondrial mRNAs and rRNAs are punctuated by tRNAs. The first stage of primary RNA processing is the excision of tRNA molecules from the polycistronic transcript, which leads to the formation of immature mRNAs and rRNAs [19,20]. This is an endonucleolytic cleavage mediated by RNAse P and elaC ribonuclease Z 2 (ELAC2), which act at the 5′ and 3′ ends of tRNAs, respectively, to release individual RNAs from polycistronic precursors [88,89]). Mitochondrial RNAse P, unlike the canonical RNase P present in the nucleus, is a complex of three proteins tRNA methyltransferase 10C (TRMT10C), hydroxysteroid 17-beta dehydrogenase 10 (HSD17B10) and protein-only RNase P catalytic subunit (PRORP) and does not contain an RNA component [88]. Released, immature transcripts undergo further processing steps or, as in the case of the majority of ncRNAs, are rapidly removed [20].

Due to the structural and transcriptional organization of the mitochondrial genome, most genes on the same strand are transcribed with equal efficiency, which leads to the formation of equal amounts of their precursors. Nevertheless, the levels of mature RNAs can differ significantly [90]. Although transcription initiated from the L-strand is more frequent than that of the H-strand [55], emerging non-coding RNAs are barely detectable [91]. These two examples show that post-transcriptional processes, especially mtRNA decay, play an important role in controlling mitochondrial gene expression to regulate steady-state levels of specific transcripts [90,92,93].

### 4.1. Degradation of mtRNAs

The machinery responsible for RNA decay in human mitochondria has remained unknown for many years. Studies performed within the last several years established that the mitochondrial degradosome, a complex of ATP-dependent RNA helicase SUPV3L1 (SUV3) and polynucleotide phosphorylase (PNPase also known as PNPT1), is a key player in mtRNA degradation [91,94]. The importance of mtRNA decay in maintaining mitochondrial homeostasis is underscored by the fact that the disruption of the SUV3 or PNPase gene is embryonically lethal in mice [95,96].

While SUV3 is a helicase that catalyzes the unwinding of RNA duplexes, an activity dependent on ATP hydrolysis by SUV3 [97], PNPase is a phosphorolytic 3′-5′ exoribonuclease, which catalyzes degradation of phosphodiester bonds in RNA [98]. In vitro experiments showed that PNPase is unable to degrade dsRNA substrates unless it forms a complex with SUV3 that unwinds the substrate for degradation [99]. The interaction between SUV3 and PNPase is a prerequisite for mtRNA degradation in vivo and occurs locally in D-foci [94]. The discovery of these structures shows that the RNA decay process in human mitochondria is spatially organized. Notably, components of the mitochondrial degradosome differ markedly in the submitochondrial localization. While SUV3 localizes only to the mitochondrial matrix [97], most of the PNPase is found in the mitochondrial intermembrane space [94,96]. Thus, only a fraction of the PNPase localizes to the mitochondrial matrix and cooperates with SUV3 in mtRNA degradation [94], whereas the rest of the PNPase functions in an SUV3-independent manner. Crystal structures of both proteins revealed the presence of some peculiarities [98,100]. Human PNPase, such as PNPases from other organisms, forms a trimer but has an untypical arrangement of RNA-binding domains [98]. Human SUV3 also has some distinctive features in terms of substrate binding, and it was even suggested that Suv3-like proteins may constitute a separate subfamily of helicases [100].

The main role of the degradosome appears to be clearing of non-coding mtRNA species, which arise mostly from transcription of the L-strand. Radiolabeling studies showed that under normal conditions, these RNAs are swiftly degraded [101]. Consequently, their steady-state levels are very low, and it is only when the degradosome function is impaired that they become readily detectable [91,94]. The degradosome complex was also found to be important in mt-mRNA turnover [94,102], 16S rRNA decay [103] and the exonucleolytic processing of the ND6 mRNA precursor [104]. The final products of the degradosome-mediated RNA decay are several nucleotides in length. These short RNA degradation intermediates are probably removed by RNA exonuclease 2 (REXO2), which is a postulated mitochondrial oligoribonuclease [105]. The activity of the mitochondrial degradosome is modulated by mtRNA binding proteins. While the complex of leucine rich pentatricopeptide repeat containing and SRA stem-loop interacting RNA binding proteins (LRPPRC-SLIRP complex) was suggested to suppress the degradosome-mediated decay of mitochondrial protein-coding RNAs [102]. G-rich RNA sequence binding factor 1 (GRSF1) was recently found to enhance the degradosome activity towards mtRNAs containing a G-quadruplex (G4), which are mostly non-coding mtRNAs [106].

### 4.2. Mitochondrial RNA-Binding Proteins (mtRBPs)

LRPPRC and SLIRP are among the best characterized noncatalytic mitochondrial RNA-binding proteins containing known RNA-interacting domains, pentatricopeptide repeats (PPR) and RRM domain, respectively. LRPPRC and SLIRP form a complex involved in the regulation of mtRNA stability, and the levels of both proteins are mutually dependent; silencing of LRPPRC results in the depletion of SLIRP and vice versa [107,108]. A recent study using RNA UV crosslinking and RNase footprinting procedures revealed that the LRPPRC-SLIRP complex modulates the secondary structures of mitochondrial transcripts, suggesting that this complex may serve as a chaperone for mtRNAs [109]. The presence of LRPPRC was shown to be important for the existence of a nontranslated, mitoribosome-unbound pool of mt-mRNAs [110]. In addition, LRPPRC was shown to be required for efficient polyadenylation of mt-mRNAs [108,110]. In mice, LRPPRC knockout is embryonically lethal [110], and in humans, mutations in the LRPPRC gene underlie Leigh syndrome, French Canadian type [111]. Recent studies suggest that LRPPRC may play a role in other various pathological states in humans, such as tumors or neurodegeneration (reviewed in detail by Cui et al. [112], emphasizing the very important role of this protein. SLIRP was shown to regulate the translation process by mediating the association of mtRNAs with mitoribosomes [113]. Surprisingly, although SLIRP knockout in mice results in extensive loss of mtRNAs, it is manifested only as a minor weight loss of the animals without any other observable phenotypes [113].

Another important RNA-binding protein, GRSF1, a member of the quasi-RRM (qRRM) family of RNA-binding proteins, was originally identified as a cytoplasmic poly(A)+ mRNA binding protein interacting with G-rich sequences [114]. Later, it was found that GRSF1 is targeted to mitochondria where it localizes to RNA-containing granules [115,116]. It was postulated that GRSF1 participates in the initial stages of polycistronic mtRNA precursor processing [116] and in the translation of some mt-mRNAs [115]. Recent findings, however, reported that GRSF1 takes part in the RNA surveillance pathway, showing that GRSF1 cooperates with the mitochondrial degradosome to regulate mtRNAs that contain G4s [106]. Vertebrates’ mitochondrial genomes have exceptional GC skews, i.e., high guanine content on one strand. As a result, transcripts that are produced by transcription of the G-poor template (i.e., L-strand) are G-rich RNAs; thus, they are prone to form G4s structures. Since G4s are stable, their presence in RNA can hinder its degradation; nevertheless, steady-state levels of mt-ncRNAs that can form G4s are extremely low. GRSF1 was found to positively regulate the degradosome-dependent decay of G4-containing mitochondrial non-coding transcripts by binding and melting G4 structures, which in turn facilitates their degradation [106,117].

Recently, two studies reported novel mtRBP engaged in the regulation of mitochondrial gene expression [81,118]. The level of C6orf203/MTRES1 protein was found to be elevated in cells under stress, and this up-regulation of MTRES1 (mitochondrial transcription rescue factor 1) was shown to prevent mitochondrial transcript loss under perturbed mitochondrial gene expression [81]. MTRES1 associates with the mitochondrial transcription machinery and acts by increasing the mitochondrial transcription without influencing the stability of mitochondrial transcripts [81]. The protective function of MTRES1 depends on its RNA-binding ability since the mutated version incapable of RNA-binding does not prevent a decrease in the mitochondrial RNA [81]. MTRES1 was also shown to associate with a large subunit of the mitochondrial ribosome and to influence mitochondrial translation [118]. Interestingly, silencing of MTRES1 causes the down-regulation of transcripts originating only from NCR without influencing other transcripts [81], which cannot explain the decrease in mitochondrial translation observed in MTRES1 knockout [118]. In contrast, it was reported that the depletion of MTRES1 leads to alterations of the mt-mRNAs’ association with the mitoribosome without influencing mitoribosome stability [118]. MTRES1 is an exciting example of mitochondrial RBP that can play a role in the regulation of mitochondrial gene expression at multiple levels. It is tempting to speculate that MTRES1 could serve as a regulatory factor coupling mitochondrial transcription and translation processes. It is possible that MTRES1 could act by interacting with mitochondrial transcription machinery and to facilitate loading of nascent mt-mRNAs on the mitoribosome. It cannot be excluded that MTRES1 could be a key regulator of mitochondrial transcription/translation coupling, especially under stress conditions. Importantly, another mtRBP, MRPL12, was shown to perform double functions in mitochondrial transcription and translation [83,84], highlighting the possible roles of MTRES1.

Another example of important players in the mitochondrial gene expression regulation concerns members of the FASTK family. Fas activated serine/threonine kinase (FASTK) and its homologs FASTKD1-5 are mitochondrially targeted RNA-binding proteins that play various roles in mtRNA metabolism as processing, translation and mitoribosome assembly proteins [104,112,119,120,121]. The FASTK family was reviewed in detail by Jourdain et al. [122]; therefore, we will not focus on these proteins here.

### 4.3. mtRNA Modifications

Mitochondrial RNAs undergo diverse modifications. One of the most common is adenylation of the 3’ end. Human mt-mRNAs, with the exception of mt-ND6, are adenylated at the 3’ ends, and this reaction is catalyzed by a noncanonical mitochondrial poly(A) polymerase (MTPAP) [123]. The role of mt-mRNAs’ adenylation is not fully understood. For some mt-mRNAs, the addition of adenine residues at the 3’ end is necessary to create a complete termination codon, as it is not encoded in the genome [21]. Initial studies reported some contradictory findings about the role of mt-mRNA polyadenylation, showing that changes in poly(A) tails had diverse effects on mt-mRNAs. Some of the transcripts were up-regulated, some were unaffected, and others were down-regulated [123,124,125,126]. This draws a speculation that individual transcripts may be differentially controlled, as it seems that adenylation may stabilize some transcripts while it may also direct other transcripts for degradation [20]. Notably, the polyadenylation pattern may vary within a cell type, and the same mt-mRNA can be adenylated to different extents in various cell types [127]. This individualized regulation is most likely achieved by transcript-specific protein-RNA interactions. In yeast, it was shown that each mt-mRNA has specific translation coactivators; moreover, it was proposed that they can function as a part of feedback control loops regulating translation efficiency according to current cell demands [128]. Similarly, the presence of the specific translation activator of cytochrome c oxidase I (TACO1) [129] was also detected in human cells. In addition to 3’-end adenylation, it was recently shown that human mt-mRNAs may also undergo other modifications, such as methylation [130,131] and pseudouridylation [132,133], unraveling an additional layer of mitochondrial gene expression regulation.

Mitochondrial tRNAs seem to be the most extensively modified among mtRNAs. Precursor tRNAs are modified at the 3’ end by tRNA nucleotidyl transferase 1 (TRNT1), which adds the CCA sequence not encoded in the genome [134]. They also undergo several other modifications, which are essential for their stability and proper function [135]. Similarly, mt-rRNAs require several chemical modifications for proper folding, stability and correct mitoribosome assembly, underscoring the important role of RNA modifications in orchestrating various mitochondrial processes [24].

The mitochondrial RNA-binding proteome has been intensely studied, and novel members of this group have been continuously discovered. Among them are mtRNA modifying enzymes, mtRNA processing and mitoribosome assembly factors. Here, we delineated only selected aspects of mtRBP-associated regulation of mitochondrial gene expression, mostly related to mtRNA degradation. For further reading on other aspects of mitochondrial RBP function and detailed insight into mt-tRNA and mt-rRNA editing as well as the mitochondrial epitranscriptome, we guide interested readers to other reviews [136,137,138].

## 5. Mitochondrial Non-Coding Transcripts and Their Implications on Human Health

### 5.1. Mitochondrial Double-Stranded RNA (mt-dsRNA) and the Innate Immune Response

Convergent transcription, synthesis of antisense RNA or expression of hairpin-containing RNAs can result in the formation of double-stranded RNA (dsRNA) [139]. RNA:RNA molecules seem to have important signaling or regulatory roles [140,141,142], and the function of a particular dsRNA depends on its source and form and the proteins that interacts with it. It is known that the presence of dsRNA can induce the antiviral response and that properly processed dsRNA can regulate gene expression [140,141,142].

The expression of the human mitochondrial genome is especially prone to produce dsRNA molecules, as this genome is an extraordinary example of convergent transcription. This results in the synthesis of complementary RNAs that can subsequently hybridize and form dsRNA. Under normal conditions, most L-strand transcripts are quickly removed [91,94], which prevents the formation of intermolecular dsRNA. Nevertheless, even in physiological conditions, mt-dsRNAs can be detected [143]. We and others have found that mtDNA transcription is a significant source of dsRNA in humans and have shown that mt-dsRNA can play a signaling role and trigger the interferon response [143,144].

The interferon response pathway is a complex process that is part of the innate immunity response, which together with the adaptive system, provides protection from pathogens. This pathway is far from being fully understood; nevertheless, it can be divided into three major steps: (1) detection of a pathogen, (2) induction of interferon (IFN) expression, and (3) up-regulation of IFN-stimulated genes. Consequently, the cell starts producing anti-pathogen agents and reshapes already active processes to combat a pathogen, or at least hampers its spread [145].

The detection step involves recognition of “pathogen-associated molecular patterns” (PAMPs), which are viral or bacterial nuclei acids, or other molecules specific for microorganisms [146]. The recognition is performed by several families of host sensors collectively called pattern recognition receptors (PRRs), such as interferon induced helicase C domain-containing protein 1 (MDA5) and RNA helicase RIG-I. The binding of immunogenic dsRNA by MDA5 and RIG-I initiates an orchestrated cascade of events that results in the up-regulation of interferon- and interferon-stimulated genes [147]. Normally, this pathway is kept silent due to the mechanisms that help to discriminate between host and foreign nucleic acids, remove potentially immunogenic host-produced nucleic acids or keep them away from PAMP receptors. For example, dsRNA, which can activate MDA5 or RIG-I, is localized in the nucleus and mitochondria, precluding its interaction with MDA5 and RIG-I, which are localized in the cytoplasm.

Recently it was found that mt-dsRNA can be released from mitochondria and, once localized to the cytoplasm, can activate interferon-dependent cellular pathways. Notably degradosome constituents SUV3 and PNPase were established as major actors in the regulation of mt-dsRNA [144]. A crucial role was assigned to PNPase in preventing the induction of the interferon response by mt-dsRNA. PNPase controls mt-dsRNA at two different levels that are spatially separated. First, as a component of the mitochondrial degradosome, it prevents the accumulation of dsRNA by degrading mitochondrial antisense transcripts. The mechanism that determines specificity of PNPase towards antisense transcripts has not been fully uncovered. This may result from targeting of PNPase-SUV3 to G4 mtRNAs by GRSF1. Alternatively, but not mutually exclusive, sense transcripts are protected by mtRBPs, while unprotected antisense mtRNAs are exposed to PNPase-SUV3. This function of PNPase requires cooperation with SUV3 and takes place in the mitochondrial matrix. A second function of PNPase in controlling mt-dsRNAs takes place in the intermembrane space, where a fraction of PNPase prevents the release of mt-dsRNA into the cytosol. This function is SUV3-independent because the helicase is absent from the intermembrane space [97]. The dysfunction of SUV3 thus results in the accumulation of mt-dsRNA exclusively in the matrix but does not lead to the release of mt-dsRNA into the cytosol due to the protective activity of PNPase in the intermembrane space. Therefore, silencing of SUV3 does not stimulate interferon expression, while an increase in interferon levels is observed in PNPase-depleted cells. Since inactivation of the degradosome components causes a vast increase in non-coding L-strand transcripts [91,94,106], it is most likely that the mt-dsRNA that accumulates upon degradosome dysfunction results from intermolecular RNA-RNA interactions. The mechanism by which mt-dsRNA exits mitochondria is unknown, and its identification will be an important step in deciphering a role of mt-dsRNA in cell biology.

The protective role of PNPase is of great importance, as revealed by the fact that mutations in *PNPT1* (PNPase encoding gene) cause pathogenesis associated with the up-regulation of mt-dsRNA and interferon response [144,148]. A comparison of the type I interferon response across species, which involved 10 mammals and one bird, revealed PNPase as one of 62 core vertebrate interferon-stimulated genes [149]. Moreover, the PNPase protein level was observed to be higher in human cells infected by human respiratory syncytial virus (HRSV), a cause of serious infections in infants [150]. Taken together with the results on the protective role of PNPase in mt-dsRNA release, these data suggest that PNPase can be involved in the suppression of the IFN response. In fact, IFN-desensitization is an important process that enables cells to recover from IFN signaling [145]. Otherwise, prolonged activation of IFN-dependent pathways can lead to permanent dysregulation of cellular homeostasis. At the organismal level, this is manifested by the development of pathologies called type I interferonopathies. An example of such a disease is Aicardi–Goutières syndrome [151].

Interestingly, the contribution of mt-dsRNA to innate immunity was also observed in other studies. It was shown that the immune response can be modulated by mt-dsRNA via protein kinase RNA-activated (PKR). The canonical PKR-induced response comprises sensing of viral dsRNA by PKR following phosphorylation of the enzyme and altering cellular signaling pathways to promote an antiviral defense. Recent findings revealed that PKR can also be induced by endogenous RNAs, primarily by mt-dsRNAs [143]. PKR was proposed to associate with mitochondria, and mt-dsRNA sensing was suggested to take part in the mitochondrial matrix [143]. However, the proposed mechanism assumes that PKR needs to be exported to the cytoplasm to induce downstream signaling pathways. How PKR translocation to and from mitochondria is maintained remains unclear. It cannot be excluded that PKR induction may take place during mt-dsRNA escape into the cytoplasm, for example, upon aberrant PNPase function.

Notably, the role of mt-dsRNA in the modulation of innate immunity is not restricted to humans, as similar responses were reported in other species, suggesting that mt-dsRNAs are important regulators of the immune response across species. A study in a fly model demonstrated that disrupted mtRNA turnover leads to mt-dsRNA accumulation and altered immune response once mt-dsRNAs are released into the cytoplasm [152]. A study conducted in a murine model showed that the lack of p53 protein results in the activation of innate immunity by dsRNA of mitochondrial origin [153]. Consistent with previous findings on human cells [144], the immune response was shown to be dependent on MDA5 and RIG-I activation [153]. Altogether, these studies established mt-dsRNAs as important regulators of the immune response and revealed a new RNA-dependent mechanism by which mitochondria regulate cell fate.

### 5.2. Mitochondrial Long Non-Coding RNAs (mt-lncRNAs)

While most non-coding transcripts are swiftly degraded after synthesis, a pool of antisense mtRNAs exhibits some stability, resulting in their detection under normal conditions. These transcripts, called mirror RNAs, such as mirror ND2 [91,94], or long non-coding RNAs (ncND5, ncND6, and ncCytB) [154], are complementary to sense transcripts. Whether these mt-lncRNAs play any role remains to be elucidated; nevertheless, differential tissue-specific expression of these RNA species [154] suggests their potential regulatory function. One can imagine that by hybridizing to their coding counterparts, mt-lncRNAs could regulate the stability or translation efficiency of corresponding mtRNAs.

Other examples of lncRNAs possibly originating from mitochondrial transcription were proposed [155,156]. An example is a 2374 nt-long transcript containing an antisense 16S mt-rRNA sequence (inverted repeat) linked to the 5′ end of the 16S mt-rRNA forming a long double-stranded structure called SncmtRNA (sense non-coding mitochondrial RNA). Interestingly, this transcript was reported to be overexpressed in proliferating but not resting tumor cells [155]. Further studies from the same group reported the presence of 2 other lncRNAs containing inverted repeats linked to the 5′ end of the antisense 16S mt-rRNA, called ASncmtRNAs (antisense non-coding mitochondrial RNAs). These transcripts were found in normal proliferating cells and were reported to be down-regulated in tumor cells [156]. Detected transcripts were postulated to be hallmarks of proliferating tumor cells vs nontumor cells, which may play a role in regulating tumor progression [156,157].

A group of lncRNAs mapped to mtDNA was detected in patients with heart disease [158], and these include circulating mitochondria-derived lncRNA LIPCAR (long intergenic non-coding RNA predicting cardiac remodeling) [159]. The 5′ end of this 781nt long chimeric lncRNA maps to the antisense of the mt-Cytb gene, while its 3′ end maps to the antisense of the mt-COX2 gene. The LIPCAR transcript was shown to be up-regulated in the late stages after myocardial infarction and to be elevated in patients with chronic heart failure, serving as a potential biomarker of cardiac remodeling and cardiovascular mortality [159,160].

**Figure 2:**
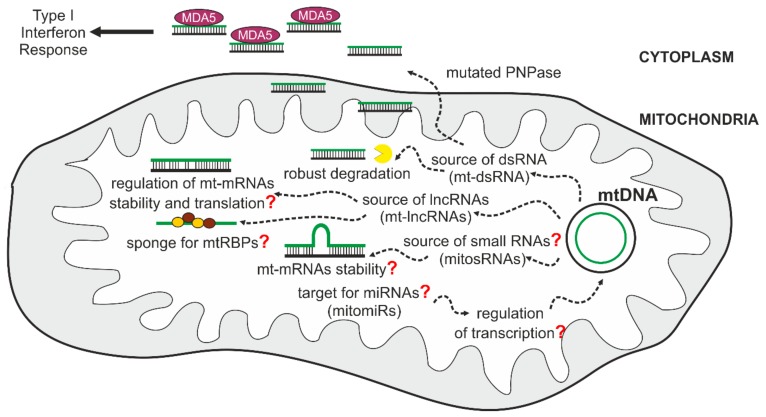
Cellular functions of mitochondria-derived transcripts. MtDNA may be a source and a target for non-coding RNA. Question mark indicates that the process or mechanism is currently undefined. Nucleus-encoded miRNAs (mitomiRs) are suggested to enter mitochondria and regulate mtDNA expression, nevertheless their exact import pathway is currently unexplained. Transcription of mtDNA may result in formation of small non-coding RNAs (mitosRNAs) which may interplay with mt-mRNAs stability. Mechanism of action of mitochondrial small non-coding RNAs is currently undefined. Long non-coding RNAs (mt-lncRNAs) may arise from mtDNA transcription and may regulate stability and transcription of mt-mRNAs or may serve as baits for mitochondrial RNA-binding proteins (mtRBPs). Convergent transcription of mtDNA may result in formation of double-stranded RNA (mt-dsRNA). Under normal conditions, L-strand derived transcripts are swiftly degraded which prevents formation of mt-dsRNAs. Dysfunction of PNPase causes accumulation of mt-dsRNA and its release into cytoplasm. Once released into the cytoplasm, mt-dsRNAs can induce type I interferon response via activation of MDA5 receptors.

As presented here, ncRNAs encompass a group of potentially important molecules that can act in various ways to influence mitochondrial function and cell physiology (Figure 2). Their putative functions involve the regulation of mitochondrial translation and mtRNA stability. They may also function as sponges occupying mtRBPs. Moreover, their ability to form mt-dsRNA together with sense transcripts predisposes them to act as signaling molecules modulating the immune response. Altogether, mitochondrial non-coding RNAs extend the repertoire of potential mechanisms by which mitochondrial gene expression is regulated and influences cell physiology. Thus far, antisense transcripts were mostly studied or linked with non-physiological conditions. It remains to be seen whether this class of RNAs has any roles under normal, nonperturbed conditions.

## 6. Emerging Aspects of mtDNA Maintenance and Expression

### 6.1. mtDNA Polymerases and mtDNA Repair

POLG, responsible for replication of mtDNA, was long believed to be the only DNA polymerase present in mitochondria [161]. In contrast, recent reports suggest that there may be more than one DNA polymerase operating in mammalian mitochondria. Recent studies proposed several additional DNA polymerases to be implicated in mtDNA metabolism; nevertheless, some of those findings require further validation [162]. The most well-documented novel mtDNA polymerase, primase and DNA directed polymerase (PrimPol), takes part both in nuclear and mitochondrial DNA maintenance [163]. PrimPol may act as a primase forming DNA and RNA primers or as a DNA polymerase to extend the DNA primers and it is also able to perform translesion synthesis [164]. It seems that in human mitochondria, the main role of PrimPol is not to prime mtDNA replication but rather to rescue stalled replication forks by bypassing lesions or repriming DNA synthesis at the site of DNA damage. Further polymerases suggested to take part in mtDNA metabolism comprise polymerases Beta (POLB), Zeta (POLZ) and Theta (POLQ) [162]. These polymerases were proposed to participate in mtDNA repair (POLB) [165] (reviewed by Kaufman and Van Houten [166], mtDNA stability (POLZ) [167] and mtDNA maintenance (POLQ) [168].

Importantly, the discovery of novel mtDNA polymerases broadens the horizon for prospective mtDNA repair pathways. Mitochondria are thought to possess a limited DNA repair system comprising mainly base excision repair (BER). In mitochondrial BER, gap filling is driven by POLG assisted by several other proteins engaged in recognition and cleavage of a damaged DNA base, elimination of the abasic site, DNA 5′-end processing to generate substrate for POLG and, finally, ligation [169,170,171,172,173,174]. Other mtDNA repair pathways are being debated; nucleotide excision repair (NER) is thought to be absent from mitochondria, and only a few studies have reported mismatch repair to be present in mitochondria [52]. It is thus possible that novel enzymes could be engaged in these processes, expanding the repertoire of possible mtDNA repair pathways. This is especially important as mtDNA damage may have significant implications for human health [175].

### 6.2. mtDNA Editing

As mitochondria are organelles separated with double membranes that harbor strict import machinery, for a long time, it has seemed impossible to stably edit mtDNA in vertebrates. While proteins can efficiently be targeted and delivered into mitochondria, nucleic acid uptake remains controversial. As such, import of the nucleic acid component for mtDNA transformation and editing appeared to be a hurdle [176]. This difficulty can be overcome by the use of protein-only nucleases that can efficiently be engineered to target the mitochondrial matrix and induce sequence-specific double-strand breaks in mtDNA molecules [177]. This strategy takes advantage of mtDNA heteroplasmy and is based on the cleavage of mtDNA molecules harboring certain sequences (for example, pathological mutations). As a double-strand break repair pathway is not present in mitochondria, cleaved mtDNA molecules are rapidly degraded [178,179,180]. Remaining, noncleaved mtDNA molecules with normal sequences can then be replicated to reconstitute mtDNA content [181,182,183,184]. Two DNA editing platforms were introduced into mitochondria: transcription activator-like effector nucleases (TALEN) [185] and zing-finger nucleases (ZFN) [183]; these platforms enabled efficient heteroplasmy shift. In 2018, two breakthrough studies published back-to-back by the Minczuk and Moraes labs showed successful mtDNA editing in vivo [186,187]. In a mouse model of heteroplasmic mitochondrial disease, mitochondrially targeted ZFN [186] and TALENs [187] delivered by the adeno-associated virus led to the successful elimination of mutated mtDNA and subsequent reversion of the pathological phenotype [186,187]. These studies may open a new era for the treatment of heteroplasmic mitochondrial diseases.

### 6.3. RNA Import into Mitochondria

As mentioned earlier, nucleic acid import into mammalian mitochondria arouses controversy. First, it is not fully clear which RNA molecules can be transported into mitochondria; second, the machinery responsible for this process is debated. PNPase, a component of the mitochondrial degradosome complex, was proposed to play a role in RNA import into mitochondria [96,188]. Indeed, a pool of PNPase is found in the mitochondrial intermembrane space [94,96,189], where it could take part in importing RNA. Nevertheless, the exact mechanism of this process is not known, and it is not certain whether PNPase plays this role alone or cooperates with other proteins. While degradation of mtRNAs is a widely accepted function of PNPase, it is unclear how the enzyme mediates the transport of RNA instead of its destruction. Another suggested pathway of RNA import concerns mitochondrial protein translocase complexes TOM and TIM [190]; however, this has not been confirmed in mammalian cells. Another controversy refers to the RNA species that could potentially be imported into mitochondria. A few studies have indicated the mitochondrial import of recombinant heterologous tRNAs comprising the introduction of motifs derived from yeast tRNA [191,192,193]. Of note, tRNAs were shown to be imported into mitochondria of trypanosomes and plants [194,195]. Nevertheless, import of endogenous RNAs H1 RNA (as an RNA component of the canonical RNaseP), 7-2 RNA (as an RNA component of the RNase MRP) and 5S rRNA (as a constituent of the mitoribosome) was recently challenged [176], as those RNAs were suggested to be dispensable in human mitochondria. Indeed, it was shown that mitochondrial RNaseP does not possess an RNA moiety [88], RNase MRP localizes predominantly to the nucleolus [196,197,198] and 5S rRNA is replaced by tRNA in the mitochondrial ribosome [199,200,201]. Establishing the molecular mechanism of mitochondrial RNA import would be of great value, as it could enable the import of Clustered Regularly Interspaced Short Palindromic Repeats (CRISPR) system constituents for mtDNA manipulation. Although obviously revolutionary and beneficial, introduction of this system in mitochondria seems to be challenging, if not impossible [176].

### 6.4. Small RNAs in Mitochondrial Biology

Transcription of mtDNA may be a source of distinctive mtRNA species. Recent studies reported that a various range of small ncRNAs may emerge form mitochondrial transcription. A study engaging high-throughput sequencing of small RNA (sRNA) revealed that around 3% of whole-cell sRNAs maps uniquely to the mitochondrial genome in 143B human cells [90]. Interestingly, most of reported unique small RNA sequences were derived from tRNA genes [90]. The functional role of these RNA species is not known and whether they can be a part of RNA interference pathway as it was shown for processed nuclear tRNAs is an open question [90,202,203]. Other reported mitochondrial sRNAs comprise small non-coding transcripts called mitosRNAs (mitochondrial genome-encoded small RNAs) [204] and mitochondrial microRNAs (also called mitomiRs) [205]. Notably, whether the novel sRNA species are just a consequence of mtRNA processing and whether they possess any biological function is currently debated. Small non-coding RNAs appear as a novel class of transcripts that may have regulatory functions in mitochondria, nevertheless their exact mode of action and some aspects of their biogenesis are far from well established and evoke controversies. A canonical function of cytoplasmic microRNA (miRNA) is sequence-specific gene silencing in RNA interference pathway [206]. Several reports suggested presence of microRNAs in mitochondria [207,208,209,210] proposed to be either products of mtDNA transcription or an effect of import of cytoplasmic transcripts inside mitochondria [205]. Nevertheless, miRNA biogenesis encompasses multiple steps of processing and require specialized machinery and it is not known which proteins could be engaged in these processes in mitochondria. Moreover, it is not clear how miRNAs could enter mitochondria. These questions need to be answered in order to establish functional potential of mitochondrial small ncRNAs.

Future studies will show whether identified short RNAs have functional importance or they are merely by-products of mtRNA processing and decay. For example, a recently described short RNA called tRNA-like accumulates strongly upon inhibition of mtRNA degradation by the mitochondrial degradosome [106]. This may imply that most, if not all, tRNA-like molecules are just by-products of mtRNA processing which are removed by the degradosome. Or this transcript has functional importance, but its levels need to be strictly controlled.

### 6.5. 37 or More?

Early studies of the human mitochondrial genome led to the mapping of mtDNA-encoded genes [21]. For a long time, human mtDNA was considered to encode 37 genes; however, a few recent reports suggested that there may be additional coding sequences within human mtDNA. Human mitochondrial genome may harbor nested small open reading frames (sORF) yielding short peptides derived from polycistronic mt-mRNAs. For example, human 16S rRNA gene was suggested to contain sORF encoding 24 amino acid peptide called humanin, which may have cytoprotective properties [211,212]. It is not known, however, whether humanin is translated inside mitochondria or in the cytoplasm and in the latter case, how humanin transcript could be exported from mitochondria and recognized by cytoplasmic translation apparatus [213]. Other mitochondria-derived peptides (MDPs) were proposed to arise from mitochondrial sORFs and were suggested to have regulatory functions within the cell. Among them are mitochondrial open reading frame of the 12S rRNA-c (MOTS-c) and small humanin-like peptides 1–6 (SHLP1-6) located within 12S rRNA and 16S rRNA genes, respectively [214,215,216]. However, synthesis of MOTS-c would require export of corresponding encoding RNA to the cytoplasm, a process of unknown mechanism. Mitochondrial sORF and sRNAs are intriguing in concept, nonetheless, initial reports of them require further substantiation. It cannot be excluded that mitochondrial sRNAs and MDPs are by-products of mitochondrial gene expression. Therefore, proposed mitochondrial sORF and sRNAs require further studies to be fully embraced by the field.

## Abbreviations

ATP	adenosine triphosphate
dsRNA	double-stranded RNA
G4	G-quadruplex
H-strand	heavy-strand
IFN	interferon
ITH	heavy-strand transcription initiation site
ITL	light-strand transcription initiation site
L-strand	light-strand
lncRNA	long non-coding RNA
MDP	mitochondria-derived peptide
miRNA	microRNA
mitomiRs	mitochondrial microRNAs
mitosRNAs	mitochondrial genome-encoded small RNAs
mtDNA	mitochondrial DNA
mtRNA	mitochondrial RNA
NCR	non-coding regulatory region
ncRNA	non-coding RNA
PAMP	pathogen-associated molecular pattern
RBP	RNA-binding protein
sORF	small Open Reading Frame
sRNA	small RNA

## References

[1] Brown G.C. (1992). Control of respiration and ATP synthesis in mammalian mitochondria and cells. Biochem. J..

[2] Go Y.-M., Jones D.P. (2008). Redox compartmentalization in eukaryotic cells. Biochim. Biophys. Acta.

[3] Titov D.V., Cracan V., Goodman R.P., Peng J., Grabarek Z., Mootha V.K. (2016). Complementation of mitochondrial electron transport chain by manipulation of the NAD+/NADH ratio. Science.

[4] Galluzzi L., Kepp O., Trojel-Hansen C., Kroemer G. (2012). Mitochondrial control of cellular life, stress, and death. Circ. Res..

[5] Pizzo P., Drago I., Filadi R., Pozzan T. (2012). Mitochondrial Ca^2+^ homeostasis: Mechanism, role, and tissue specificities. Pflugers Arch..

[6] Brand M.D., Orr A.L., Perevoshchikova I.V., Quinlan C.L. (2013). The role of mitochondrial function and cellular bioenergetics in ageing and disease. Br. J. Dermatol..

[7] Maiese K. (2016). The bright side of reactive oxygen species: Lifespan extension without cellular demise. J. Transl. Sci..

[8] Arnoult D., Soares F., Tattoli I., Girardin S.E. (2011). Mitochondria in innate immunity. EMBO Rep..

[9] Sandhir R., Halder A., Sunkaria A. (2017). Mitochondria as a centrally positioned hub in the innate immune response. Biochim. Biophys. Acta Mol. Basis Dis..

[10] Czarnecka A.M., Golik P., Bartnik E. (2006). Mitochondrial DNA mutations in human neoplasia. J. Appl. Genet..

[11] Lin M.T., Beal M.F. (2006). Mitochondrial dysfunction and oxidative stress in neurodegenerative diseases. Nature.

[12] Copeland W.C., Wachsman J.T., Johnson F.M., Penta J.S. (2002). Mitochondrial DNA alterations in cancer. Cancer Invest..

[13] Schapira A.H.V. (2012). Mitochondrial diseases. Lancet.

[14] Nicholls T.J., Rorbach J., Minczuk M. (2013). Mitochondria: Mitochondrial RNA metabolism and human disease. Int. J. Biochem. Cell Biol..

[15] Vafai S.B., Mootha V.K. (2012). Mitochondrial disorders as windows into an ancient organelle. Nature.

[16] Gorman G.S., Chinnery P.F., DiMauro S., Hirano M., Koga Y., McFarland R., Suomalainen A., Thorburn D.R., Zeviani M., Turnbull D.M. (2016). Mitochondrial diseases. Nat. Rev. Dis. Primers.

[17] Rusecka J., Kaliszewska M., Bartnik E., Tońska K. (2018). Nuclear genes involved in mitochondrial diseases caused by instability of mitochondrial DNA. J. Appl. Genet..

[18] Taanman J.W. (1999). The mitochondrial genome: Structure, transcription, translation and replication. Biochim. Biophys. Acta.

[19] Fernández-Silva P., Enriquez J.A., Montoya J. (2003). Replication and transcription of mammalian mitochondrial DNA. Exp. Physiol..

[20] Rorbach J., Minczuk M. (2012). The post-transcriptional life of mammalian mitochondrial RNA. Biochem. J..

[21] Anderson S., Bankier A.T., Barrell B.G., de Bruijn M.H., Coulson A.R., Drouin J., Eperon I.C., Nierlich D.P., Roe B.A., Sanger F. (1981). Sequence and organization of the human mitochondrial genome. Nature.

[22] Calvo S.E., Clauser K.R., Mootha V.K. (2016). MitoCarta2.0: An updated inventory of mammalian mitochondrial proteins. Nucleic Acids Res..

[23] Schmidt O., Pfanner N., Meisinger C. (2010). Mitochondrial protein import: From proteomics to functional mechanisms. Nat. Rev. Mol. Cell Biol..

[24] Van Haute L., Pearce S.F., Powell C.A., D’Souza A.R., Nicholls T.J., Minczuk M. (2015). Mitochondrial transcript maturation and its disorders. J. Inherit. Metab. Dis..

[25] Yang D., Oyaizu Y., Oyaizu H., Olsen G.J., Woese C.R. (1985). Mitochondrial origins. Proc. Natl. Acad. Sci. USA.

[26] Gray M.W., Burger G., Lang B.F. (1999). Mitochondrial evolution. Science.

[27] Martijn J., Vosseberg J., Guy L., Offre P., Ettema T.J.G. (2018). Deep mitochondrial origin outside the sampled alphaproteobacteria. Nature.

[28] Nass M.M., Nass S. (1963). Intramitochondrial fibers with DNA characteristics. I. fixation and electron staining reactions. J. Cell Biol..

[29] Anderson S. (1981). Shotgun DNA sequencing using cloned DNase I-generated fragments. Nucleic Acids Res..

[30] Bonekamp N.A., Larsson N.-G. (2018). SnapShot: Mitochondrial Nucleoid. Cell.

[31] Garrido N., Griparic L., Jokitalo E., Wartiovaara J., van der Bliek A.M., Spelbrink J.N. (2003). Composition and dynamics of human mitochondrial nucleoids. Mol. Biol. Cell.

[32] Wang Y., Bogenhagen D.F. (2006). Human mitochondrial DNA nucleoids are linked to protein folding machinery and metabolic enzymes at the mitochondrial inner membrane. J. Biol. Chem..

[33] Kukat C., Wurm C.A., Spåhr H., Falkenberg M., Larsson N.-G., Jakobs S. (2011). Super-resolution microscopy reveals that mammalian mitochondrial nucleoids have a uniform size and frequently contain a single copy of mtDNA. Proc. Natl. Acad. Sci. USA.

[34] Kukat C., Davies K.M., Wurm C.A., Spåhr H., Bonekamp N.A., Kühl I., Joos F., Polosa P.L., Park C.B., Posse V. (2015). Cross-strand binding of TFAM to a single mtDNA molecule forms the mitochondrial nucleoid. Proc. Natl. Acad. Sci. USA.

[35] Ekstrand M.I., Falkenberg M., Rantanen A., Park C.B., Gaspari M., Hultenby K., Rustin P., Gustafsson C.M., Larsson N.-G. (2004). Mitochondrial transcription factor A regulates mtDNA copy number in mammals. Hum. Mol. Genet..

[36] Satoh M., Kuroiwa T. (1991). Organization of multiple nucleoids and DNA molecules in mitochondria of a human cell. Exp. Cell Res..

[37] Legros F., Malka F., Frachon P., Lombès A., Rojo M. (2004). Organization and dynamics of human mitochondrial DNA. J. Cell Sci..

[38] Iborra F.J., Kimura H., Cook P.R. (2004). The functional organization of mitochondrial genomes in human cells. BMC Biol..

[39] Jenuth J.P., Peterson A.C., Fu K., Shoubridge E.A. (1996). Random genetic drift in the female germline explains the rapid segregation of mammalian mitochondrial DNA. Nat. Genet..

[40] Ishihara T., Ban-Ishihara R., Maeda M., Matsunaga Y., Ichimura A., Kyogoku S., Aoki H., Katada S., Nakada K., Nomura M. (2015). Dynamics of mitochondrial DNA nucleoids regulated by mitochondrial fission is essential for maintenance of homogeneously active mitochondria during neonatal heart development. Mol. Cell. Biol..

[41] Kvist L., Martens J., Nazarenko A.A., Orell M. (2003). Paternal leakage of mitochondrial DNA in the great tit (Parus major). Mol. Biol. Evol..

[42] Cree L.M., Samuels D.C., de Sousa Lopes S.C., Rajasimha H.K., Wonnapinij P., Mann J.R., Dahl H.-H.M., Chinnery P.F. (2008). A reduction of mitochondrial DNA molecules during embryogenesis explains the rapid segregation of genotypes. Nat. Genet..

[43] Shoubridge E.A. (2000). Mitochondrial DNA segregation in the developing embryo. Hum. Reprod..

[44] Schwartz M., Vissing J. (2002). Paternal inheritance of mitochondrial DNA. N. Engl. J. Med..

[45] Luo S., Valencia C.A., Zhang J., Lee N.-C., Slone J., Gui B., Wang X., Li Z., Dell S., Brown J. (2018). Biparental Inheritance of Mitochondrial DNA in Humans. Proc. Natl. Acad. Sci. USA.

[46] Pohjoismäki J.L.O., Wanrooij S., Hyvärinen A.K., Goffart S., Holt I.J., Spelbrink J.N., Jacobs H.T. (2006). Alterations to the expression level of mitochondrial transcription factor A, TFAM, modify the mode of mitochondrial DNA replication in cultured human cells. Nucleic Acids Res..

[47] Pohjoismäki J.L.O., Goffart S., Tyynismaa H., Willcox S., Ide T., Kang D., Suomalainen A., Karhunen P.J., Griffith J.D., Holt I.J. (2009). Human heart mitochondrial DNA is organized in complex catenated networks containing abundant four-way junctions and replication forks. J. Biol. Chem..

[48] Tuppen H.A.L., Blakely E.L., Turnbull D.M., Taylor R.W. (2010). Mitochondrial DNA mutations and human disease. Biochim. Biophys. Acta.

[49] Holt I.J., Reyes A. (2012). Human mitochondrial DNA replication. Cold Spring Harb. Perspect. Biol..

[50] Gustafsson C.M., Falkenberg M., Larsson N.-G. (2016). Maintenance and Expression of Mammalian Mitochondrial DNA. Annu. Rev. Biochem..

[51] Young M.J., Copeland W.C. (2016). Human mitochondrial DNA replication machinery and disease. Curr. Opin. Genet. Dev..

[52] Copeland W.C., Longley M.J. (2014). Mitochondrial genome maintenance in health and disease. DNA Repair.

[53] Attardi G., Chomyn A., King M.P., Kruse B., Polosa P.L., Murdter N.N. (1990). Regulation of mitochondrial gene expression in mammalian cells. Biochem. Soc. Trans..

[54] Fish J., Raule N., Attardi G. (2004). Discovery of a major D-loop replication origin reveals two modes of human mtDNA synthesis. Science.

[55] Aloni Y., Attardi G. (1971). Expression of the mitochondrial genome in HeLa cells. II. Evidence for complete transcription of mitochondrial DNA. J. Mol. Biol..

[56] Hillen H.S., Temiakov D., Cramer P. (2018). Structural basis of mitochondrial transcription. Nat. Struct. Mol. Biol..

[57] Fisher R.P., Clayton D.A. (1985). A transcription factor required for promoter recognition by human mitochondrial RNA polymerase. Accurate initiation at the heavy- and light-strand promoters dissected and reconstituted in vitro. J. Biol. Chem..

[58] Morozov Y.I., Parshin A.V., Agaronyan K., Cheung A.C.M., Anikin M., Cramer P., Temiakov D. (2015). A model for transcription initiation in human mitochondria. Nucleic Acids Res..

[59] Hillen H.S., Morozov Y.I., Sarfallah A., Temiakov D., Cramer P. (2017). Structural Basis of Mitochondrial Transcription Initiation. Cell.

[60] Minczuk M., He J., Duch A.M., Ettema T.J., Chlebowski A., Dzionek K., Nijtmans L.G.J., Huynen M.A., Holt I.J. (2011). TEFM (c17orf42) is necessary for transcription of human mtDNA. Nucleic Acids Res..

[61] Posse V., Shahzad S., Falkenberg M., Hällberg B.M., Gustafsson C.M. (2015). TEFM is a potent stimulator of mitochondrial transcription elongation in vitro. Nucleic Acids Res..

[62] Yu H., Xue C., Long M., Jia H., Xue G., Du S., Coello Y., Ishibashi T. (2018). TEFM Enhances Transcription Elongation by Modifying mtRNAP Pausing Dynamics. Biophys. J..

[63] Agaronyan K., Morozov Y.I., Anikin M., Temiakov D. (2015). Mitochondrial biology. Replication-transcription switch in human mitochondria. Science.

[64] Hillen H.S., Parshin A.V., Agaronyan K., Morozov Y.I., Graber J.J., Chernev A., Schwinghammer K., Urlaub H., Anikin M., Cramer P. (2017). Mechanism of Transcription Anti-termination in Human Mitochondria. Cell.

[65] Jiang S., Koolmeister C., Misic J., Siira S., Kühl I., Silva Ramos E., Miranda M., Jiang M., Posse V., Lytovchenko O. (2019). TEFM regulates both transcription elongation and RNA processing in mitochondria. EMBO Rep..

[66] Kruse B., Narasimhan N., Attardi G. (1989). Termination of transcription in human mitochondria: Identification and purification of a DNA binding protein factor that promotes termination. Cell.

[67] Yakubovskaya E., Mejia E., Byrnes J., Hambardjieva E., Garcia-Diaz M. (2010). Helix unwinding and base flipping enable human MTERF1 to terminate mitochondrial transcription. Cell.

[68] Terzioglu M., Ruzzenente B., Harmel J., Mourier A., Jemt E., López M.D., Kukat C., Stewart J.B., Wibom R., Meharg C. (2013). MTERF1 binds mtDNA to prevent transcriptional interference at the light-strand promoter but is dispensable for rRNA gene transcription regulation. Cell Metab..

[69] Shi Y., Posse V., Zhu X., Hyvärinen A.K., Jacobs H.T., Falkenberg M., Gustafsson C.M. (2016). Mitochondrial transcription termination factor 1 directs polar replication fork pausing. Nucleic Acids Res..

[70] Linder T., Park C.B., Asin-Cayuela J., Pellegrini M., Larsson N.-G., Falkenberg M., Samuelsson T., Gustafsson C.M. (2005). A family of putative transcription termination factors shared amongst metazoans and plants. Curr. Genet..

[71] Pellegrini M., Asin-Cayuela J., Erdjument-Bromage H., Tempst P., Larsson N.-G., Gustafsson C.M. (2009). MTERF2 is a nucleoid component in mammalian mitochondria. Biochim. Biophys. Acta.

[72] Cámara Y., Asin-Cayuela J., Park C.B., Metodiev M.D., Shi Y., Ruzzenente B., Kukat C., Habermann B., Wibom R., Hultenby K. (2011). MTERF4 regulates translation by targeting the methyltransferase NSUN4 to the mammalian mitochondrial ribosome. Cell Metab..

[73] Wredenberg A., Lagouge M., Bratic A., Metodiev M.D., Spåhr H., Mourier A., Freyer C., Ruzzenente B., Tain L., Grönke S. (2013). MTERF3 regulates mitochondrial ribosome biogenesis in invertebrates and mammals. PLoS Genet..

[74] Alam T.I., Kanki T., Muta T., Ukaji K., Abe Y., Nakayama H., Takio K., Hamasaki N., Kang D. (2003). Human mitochondrial DNA is packaged with TFAM. Nucleic Acids Res..

[75] Kaufman B.A., Durisic N., Mativetsky J.M., Costantino S., Hancock M.A., Grutter P., Shoubridge E.A. (2007). The mitochondrial transcription factor TFAM coordinates the assembly of multiple DNA molecules into nucleoid-like structures. Mol. Biol. Cell.

[76] Shadel G.S., Clayton D.A. (1997). Mitochondrial DNA maintenance in vertebrates. Annu. Rev. Biochem..

[77] Campbell C.T., Kolesar J.E., Kaufman B.A. (2012). Mitochondrial transcription factor A regulates mitochondrial transcription initiation, DNA packaging, and genome copy number. Biochim. Biophys. Acta.

[78] Larsson N.G., Wang J., Wilhelmsson H., Oldfors A., Rustin P., Lewandoski M., Barsh G.S., Clayton D.A. (1998). Mitochondrial transcription factor A is necessary for mtDNA maintenance and embryogenesis in mice. Nat. Genet..

[79] Stiles A.R., Simon M.T., Stover A., Eftekharian S., Khanlou N., Wang H.L., Magaki S., Lee H., Partynski K., Dorrani N. (2016). Mutations in TFAM, encoding mitochondrial transcription factor A, cause neonatal liver failure associated with mtDNA depletion. Mol. Genet. Metab..

[80] Kang I., Chu C.T., Kaufman B.A. (2018). The mitochondrial transcription factor TFAM in neurodegeneration: Emerging evidence and mechanisms. FEBS Lett..

[81] Kotrys A.V., Cysewski D., Czarnomska S.D., Pietras Z., Borowski L.S., Dziembowski A., Szczesny R.J. (2019). Quantitative proteomics revealed C6orf203/MTRES1 as a factor preventing stress-induced transcription deficiency in human mitochondria. Nucleic Acids Res..

[82] Serre V., Rozanska A., Beinat M., Chretien D., Boddaert N., Munnich A., Rötig A., Chrzanowska-Lightowlers Z.M. (2013). Mutations in mitochondrial ribosomal protein MRPL12 leads to growth retardation, neurological deterioration and mitochondrial translation deficiency. Biochim. Biophys. Acta.

[83] Surovtseva Y.V., Shutt T.E., Cotney J., Cimen H., Chen S.Y., Koc E.C., Shadel G.S. (2011). Mitochondrial ribosomal protein L12 selectively associates with human mitochondrial RNA polymerase to activate transcription. Proc. Natl. Acad. Sci. USA.

[84] Wang Z., Cotney J., Shadel G.S. (2007). Human mitochondrial ribosomal protein MRPL12 interacts directly with mitochondrial RNA polymerase to modulate mitochondrial gene expression. J. Biol. Chem..

[85] Nouws J., Goswami A.V., Bestwick M., McCann B.J., Surovtseva Y.V., Shadel G.S. (2016). Mitochondrial Ribosomal Protein L12 Is Required for POLRMT Stability and Exists as Two Forms Generated by Alternative Proteolysis during Import. J. Biol. Chem..

[86] Barchiesi A., Vascotto C. (2019). Transcription, Processing, and Decay of Mitochondrial RNA in Health and Disease. Int. J. Mol. Sci..

[87] Ojala D., Montoya J., Attardi G. (1981). tRNA punctuation model of RNA processing in human mitochondria. Nature.

[88] Holzmann J., Frank P., Löffler E., Bennett K.L., Gerner C., Rossmanith W. (2008). RNase P without RNA: Identification and functional reconstitution of the human mitochondrial tRNA processing enzyme. Cell.

[89] Brzezniak L.K., Bijata M., Szczesny R.J., Stepien P.P. (2011). Involvement of human ELAC2 gene product in 3’ end processing of mitochondrial tRNAs. RNA Biol..

[90] Mercer T.R., Neph S., Dinger M.E., Crawford J., Smith M.A., Shearwood A.-M.J., Haugen E., Bracken C.P., Rackham O., Stamatoyannopoulos J.A. (2011). The human mitochondrial transcriptome. Cell.

[91] Szczesny R.J., Borowski L.S., Brzezniak L.K., Dmochowska A., Gewartowski K., Bartnik E., Stepien P.P. (2010). Human mitochondrial RNA turnover caught in flagranti: Involvement of hSuv3p helicase in RNA surveillance. Nucleic Acids Res..

[92] Piechota J., Tomecki R., Gewartowski K., Szczesny R., Dmochowska A., Kudła M., Dybczyńska L., Stepien P.P., Bartnik E. (2006). Differential stability of mitochondrial mRNA in HeLa cells. Acta Biochim. Pol..

[93] Szczesny R.J., Borowski L.S., Malecki M., Wojcik M.A., Stepien P.P., Golik P. (2012). RNA degradation in yeast and human mitochondria. Biochim. Biophys. Acta.

[94] Borowski L.S., Dziembowski A., Hejnowicz M.S., Stepien P.P., Szczesny R.J. (2013). Human mitochondrial RNA decay mediated by PNPase-hSuv3 complex takes place in distinct foci. Nucleic Acids Res..

[95] Pereira M., Mason P., Szczesny R.J., Maddukuri L., Dziwura S., Jedrzejczak R., Paul E., Wojcik A., Dybczynska L., Tudek B. (2007). Interaction of human SUV3 RNA/DNA helicase with BLM helicase; loss of the SUV3 gene results in mouse embryonic lethality. Mech. Ageing Dev..

[96] Wang G., Chen H.-W., Oktay Y., Zhang J., Allen E.L., Smith G.M., Fan K.C., Hong J.S., French S.W., McCaffery J.M. (2010). PNPASE regulates RNA import into mitochondria. Cell.

[97] Minczuk M., Piwowarski J., Papworth M.A., Awiszus K., Schalinski S., Dziembowski A., Dmochowska A., Bartnik E., Tokatlidis K., Stepien P.P. (2002). Localisation of the human hSuv3p helicase in the mitochondrial matrix and its preferential unwinding of dsDNA. Nucleic Acids Res..

[98] Lin C.L., Wang Y.-T., Yang W.-Z., Hsiao Y.-Y., Yuan H.S. (2012). Crystal structure of human polynucleotide phosphorylase: Insights into its domain function in RNA binding and degradation. Nucleic Acids Res..

[99] Wang D.D.-H., Shu Z., Lieser S.A., Chen P.-L., Lee W.-H. (2009). Human mitochondrial SUV3 and polynucleotide phosphorylase form a 330-kDa heteropentamer to cooperatively degrade double-stranded RNA with a 3’-to-5’ directionality. J. Biol. Chem..

[100] Jedrzejczak R., Wang J., Dauter M., Szczesny R.J., Stepien P.P., Dauter Z. (2011). Human Suv3 protein reveals unique features among SF2 helicases. Acta Crystallogr. D Biol. Crystallogr..

[101] Aloni Y., Attardi G. (1971). Symmetrical in vivo transcription of mitochondrial DNA in HeLa cells. Proc. Natl. Acad. Sci. USA.

[102] Chujo T., Ohira T., Sakaguchi Y., Goshima N., Nomura N., Nagao A., Suzuki T. (2012). LRPPRC/SLIRP suppresses PNPase-mediated mRNA decay and promotes polyadenylation in human mitochondria. Nucleic Acids Res..

[103] Tu Y.-T., Barrientos A. (2015). The Human Mitochondrial DEAD-Box Protein DDX28 Resides in RNA Granules and Functions in Mitoribosome Assembly. Cell Rep..

[104] Jourdain A.A., Koppen M., Rodley C.D., Maundrell K., Gueguen N., Reynier P., Guaras A.M., Enriquez J.A., Anderson P., Simarro M. (2015). A mitochondria-specific isoform of FASTK is present in mitochondrial RNA granules and regulates gene expression and function. Cell Rep..

[105] Bruni F., Gramegna P., Oliveira J.M.A., Lightowlers R.N., Chrzanowska-Lightowlers Z.M.A. (2013). REXO2 is an oligoribonuclease active in human mitochondria. PLoS ONE.

[106] Pietras Z., Wojcik M.A., Borowski L.S., Szewczyk M., Kulinski T.M., Cysewski D., Stepien P.P., Dziembowski A., Szczesny R.J. (2018). Dedicated surveillance mechanism controls G-quadruplex forming non-coding RNAs in human mitochondria. Nat. Commun..

[107] Baughman J.M., Nilsson R., Gohil V.M., Arlow D.H., Gauhar Z., Mootha V.K. (2009). A computational screen for regulators of oxidative phosphorylation implicates SLIRP in mitochondrial RNA homeostasis. PLoS Genet..

[108] Sasarman F., Brunel-Guitton C., Antonicka H., Wai T., Shoubridge E.A. (2010). LSFC Consortium LRPPRC and SLIRP interact in a ribonucleoprotein complex that regulates posttranscriptional gene expression in mitochondria. Mol. Biol. Cell.

[109] Siira S.J., Spåhr H., Shearwood A.-M.J., Ruzzenente B., Larsson N.-G., Rackham O., Filipovska A. (2017). LRPPRC-mediated folding of the mitochondrial transcriptome. Nat. Commun..

[110] Ruzzenente B., Metodiev M.D., Wredenberg A., Bratic A., Park C.B., Cámara Y., Milenkovic D., Zickermann V., Wibom R., Hultenby K. (2012). LRPPRC is necessary for polyadenylation and coordination of translation of mitochondrial mRNAs. EMBO J..

[111] Mootha V.K., Lepage P., Miller K., Bunkenborg J., Reich M., Hjerrild M., Delmonte T., Villeneuve A., Sladek R., Xu F. (2003). Identification of a gene causing human cytochrome c oxidase deficiency by integrative genomics. Proc. Natl. Acad. Sci. USA.

[112] Cui J., Wang L., Ren X., Zhang Y., Zhang H. (2019). LRPPRC: A Multifunctional Protein Involved in Energy Metabolism and Human Disease. Front. Physiol..

[113] Lagouge M., Mourier A., Lee H.J., Spåhr H., Wai T., Kukat C., Silva Ramos E., Motori E., Busch J.D., Siira S. (2015). SLIRP Regulates the Rate of Mitochondrial Protein Synthesis and Protects LRPPRC from Degradation. PLoS Genet..

[114] Qian Z., Wilusz J. (1994). GRSF-1: A poly(A)+ mRNA binding protein which interacts with a conserved G-rich element. Nucleic Acids Res..

[115] Antonicka H., Sasarman F., Nishimura T., Paupe V., Shoubridge E.A. (2013). The mitochondrial RNA-binding protein GRSF1 localizes to RNA granules and is required for posttranscriptional mitochondrial gene expression. Cell Metab..

[116] Jourdain A.A., Koppen M., Wydro M., Rodley C.D., Lightowlers R.N., Chrzanowska-Lightowlers Z.M., Martinou J.-C. (2013). GRSF1 regulates RNA processing in mitochondrial RNA granules. Cell Metab..

[117] Pietras Z., Wojcik M.A., Borowski L.S., Szewczyk M., Kulinski T.M., Cysewski D., Stepien P.P., Dziembowski A., Szczesny R.J. (2018). Controlling the mitochondrial antisense—role of the SUV3-PNPase complex and its co-factor GRSF1 in mitochondrial RNA surveillance. Mol. Cell. Oncol..

[118] Gopalakrishna S., Pearce S.F., Dinan A.M., Schober F.A., Cipullo M., Spåhr H., Khawaja A., Maffezzini C., Freyer C., Wredenberg A. (2019). C6orf203 is an RNA-binding protein involved in mitochondrial protein synthesis. Nucleic Acids Res..

[119] Simarro M., Gimenez-Cassina A., Kedersha N., Lazaro J.-B., Adelmant G.O., Marto J.A., Rhee K., Tisdale S., Danial N., Benarafa C. (2010). Fast kinase domain-containing protein 3 is a mitochondrial protein essential for cellular respiration. Biochem. Biophys. Res. Commun..

[120] Antonicka H., Shoubridge E.A. (2015). Mitochondrial RNA Granules Are Centers for Posttranscriptional RNA Processing and Ribosome Biogenesis. Cell Rep..

[121] Popow J., Alleaume A.-M., Curk T., Schwarzl T., Sauer S., Hentze M.W. (2015). FASTKD2 is an RNA-binding protein required for mitochondrial RNA processing and translation. RNA.

[122] Jourdain A.A., Popow J., de la Fuente M.A., Martinou J.-C., Anderson P., Simarro M. (2017). The FASTK family of proteins: Emerging regulators of mitochondrial RNA biology. Nucleic Acids Res..

[123] Tomecki R., Dmochowska A., Gewartowski K., Dziembowski A., Stepien P.P. (2004). Identification of a novel human nuclear-encoded mitochondrial poly(A) polymerase. Nucleic Acids Res..

[124] Nagaike T., Suzuki T., Katoh T., Ueda T. (2005). Human mitochondrial mRNAs are stabilized with polyadenylation regulated by mitochondria-specific poly(A) polymerase and polynucleotide phosphorylase. J. Biol. Chem..

[125] Wydro M., Bobrowicz A., Temperley R.J., Lightowlers R.N., Chrzanowska-Lightowlers Z.M. (2010). Targeting of the cytosolic poly(A) binding protein PABPC1 to mitochondria causes mitochondrial translation inhibition. Nucleic Acids Res..

[126] Rorbach J., Nicholls T.J.J., Minczuk M. (2011). PDE12 removes mitochondrial RNA poly(A) tails and controls translation in human mitochondria. Nucleic Acids Res..

[127] Temperley R.J., Wydro M., Lightowlers R.N., Chrzanowska-Lightowlers Z.M. (2010). Human mitochondrial mRNAs--like members of all families, similar but different. Biochim. Biophys. Acta.

[128] Herrmann J.M., Woellhaf M.W., Bonnefoy N. (2013). Control of protein synthesis in yeast mitochondria: The concept of translational activators. Biochim. Biophys. Acta.

[129] Weraarpachai W., Antonicka H., Sasarman F., Seeger J., Schrank B., Kolesar J.E., Lochmüller H., Chevrette M., Kaufman B.A., Horvath R. (2009). Mutation in TACO1, encoding a translational activator of COX I, results in cytochrome c oxidase deficiency and late-onset Leigh syndrome. Nat. Genet..

[130] Safra M., Sas-Chen A., Nir R., Winkler R., Nachshon A., Bar-Yaacov D., Erlacher M., Rossmanith W., Stern-Ginossar N., Schwartz S. (2017). The m1A landscape on cytosolic and mitochondrial mRNA at single-base resolution. Nature.

[131] Li X., Xiong X., Zhang M., Wang K., Chen Y., Zhou J., Mao Y., Lv J., Yi D., Chen X.-W. (2017). Base-Resolution Mapping Reveals Distinct m1A Methylome in Nuclear- and Mitochondrial-Encoded Transcripts. Mol. Cell.

[132] Carlile T.M., Rojas-Duran M.F., Zinshteyn B., Shin H., Bartoli K.M., Gilbert W.V. (2014). Pseudouridine profiling reveals regulated mRNA pseudouridylation in yeast and human cells. Nature.

[133] Li X., Zhu P., Ma S., Song J., Bai J., Sun F., Yi C. (2015). Chemical pulldown reveals dynamic pseudouridylation of the mammalian transcriptome. Nat. Chem. Biol..

[134] Nagaike T., Suzuki T., Tomari Y., Takemoto-Hori C., Negayama F., Watanabe K., Ueda T. (2001). Identification and characterization of mammalian mitochondrial tRNA nucleotidyltransferases. J. Biol. Chem..

[135] Suzuki T., Nagao A., Suzuki T. (2011). Human mitochondrial tRNAs: Biogenesis, function, structural aspects, and diseases. Annu. Rev. Genet..

[136] Rackham O., Mercer T.R., Filipovska A. (2012). The human mitochondrial transcriptome and the RNA-binding proteins that regulate its expression. Wiley Interdiscip. Rev. RNA.

[137] Pearce S.F., Rebelo-Guiomar P., D’Souza A.R., Powell C.A., Van Haute L., Minczuk M. (2017). Regulation of Mammalian Mitochondrial Gene Expression: Recent Advances. Trends Biochem. Sci..

[138] Lee R.G., Rudler D.L., Rackham O., Filipovska A. (2018). Is mitochondrial gene expression coordinated or stochastic?. Biochem. Soc. Trans..

[139] White E., Schlackow M., Kamieniarz-Gdula K., Proudfoot N.J., Gullerova M. (2014). Human nuclear Dicer restricts the deleterious accumulation of endogenous double-stranded RNA. Nat. Struct. Mol. Biol..

[140] Gantier M.P. (2014). Processing of double-stranded RNA in mammalian cells: A direct antiviral role?. J. Interferon Cytokine Res..

[141] Sen G.C. (2014). Biological functions of double-stranded RNA and its binding proteins. J. Interferon Cytokine Res..

[142] Svoboda P. (2014). Renaissance of mammalian endogenous RNAi. FEBS Lett..

[143] Kim Y., Park J., Kim S., Kim M., Kang M.-G., Kwak C., Kang M., Kim B., Rhee H.-W., Kim V.N. (2018). PKR Senses Nuclear and Mitochondrial Signals by Interacting with Endogenous Double-Stranded RNAs. Mol. Cell.

[144] Dhir A., Dhir S., Borowski L.S., Jimenez L., Teitell M., Rötig A., Crow Y.J., Rice G.I., Duffy D., Tamby C. (2018). Mitochondrial double-stranded RNA triggers antiviral signalling in humans. Nature.

[145] Schneider W.M., Chevillotte M.D., Rice C.M. (2014). Interferon-stimulated genes: A complex web of host defenses. Annu. Rev. Immunol..

[146] Medzhitov R. (2007). Recognition of microorganisms and activation of the immune response. Nature.

[147] Belgnaoui S.M., Paz S., Hiscott J. (2011). Orchestrating the interferon antiviral response through the mitochondrial antiviral signaling (MAVS) adapter. Curr. Opin. Immunol..

[148] Rius R., Van Bergen N.J., Compton A.G., Riley L.G., Kava M.P., Balasubramaniam S., Amor D.J., Fanjul-Fernandez M., Cowley M.J., Fahey M.C. (2019). Clinical Spectrum and Functional Consequences Associated with Bi-Allelic Pathogenic PNPT1 Variants. J. Clin. Med..

[149] Shaw A.E., Hughes J., Gu Q., Behdenna A., Singer J.B., Dennis T., Orton R.J., Varela M., Gifford R.J., Wilson S.J. (2017). Fundamental properties of the mammalian innate immune system revealed by multispecies comparison of type I interferon responses. PLoS Biol..

[150] Munday D.C., Hiscox J.A., Barr J.N. (2010). Quantitative proteomic analysis of A549 cells infected with human respiratory syncytial virus subgroup B using SILAC coupled to LC-MS/MS. Proteomics.

[151] Crow Y.J., Manel N. (2015). Aicardi-Goutières syndrome and the type I interferonopathies. Nat. Rev. Immunol..

[152] Pajak A., Laine I., Clemente P., El-Fissi N., Schober F.A., Maffezzini C., Calvo-Garrido J., Wibom R., Filograna R., Dhir A. (2019). Defects of mitochondrial RNA turnover lead to the accumulation of double-stranded RNA in vivo. PLoS Genet..

[153] Wiatrek D.M., Candela M.E., Sedmík J., Oppelt J., Keegan L.P., O’Connell M.A. (2019). Activation of innate immunity by mitochondrial dsRNA in mouse cells lacking p53 protein. RNA.

[154] Rackham O., Shearwood A.-M.J., Mercer T.R., Davies S.M.K., Mattick J.S., Filipovska A. (2011). Long noncoding RNAs are generated from the mitochondrial genome and regulated by nuclear-encoded proteins. RNA.

[155] Villegas J., Burzio V., Villota C., Landerer E., Martinez R., Santander M., Martinez R., Pinto R., Vera M.I., Boccardo E. (2007). Expression of a novel non-coding mitochondrial RNA in human proliferating cells. Nucleic Acids Res..

[156] Burzio V.A., Villota C., Villegas J., Landerer E., Boccardo E., Villa L.L., Martínez R., Lopez C., Gaete F., Toro V. (2009). Expression of a family of noncoding mitochondrial RNAs distinguishes normal from cancer cells. Proc. Natl. Acad. Sci. USA.

[157] Vidaurre S., Fitzpatrick C., Burzio V.A., Briones M., Villota C., Villegas J., Echenique J., Oliveira-Cruz L., Araya M., Borgna V. (2014). Down-regulation of the antisense mitochondrial non-coding RNAs (ncRNAs) is a unique vulnerability of cancer cells and a potential target for cancer therapy. J. Biol. Chem..

[158] Yang K.-C., Yamada K.A., Patel A.Y., Topkara V.K., George I., Cheema F.H., Ewald G.A., Mann D.L., Nerbonne J.M. (2014). Deep RNA sequencing reveals dynamic regulation of myocardial noncoding RNAs in failing human heart and remodeling with mechanical circulatory support. Circulation.

[159] Kumarswamy R., Bauters C., Volkmann I., Maury F., Fetisch J., Holzmann A., Lemesle G., de Groote P., Pinet F., Thum T. (2014). Circulating long noncoding RNA, LIPCAR, predicts survival in patients with heart failure. Circ. Res..

[160] Dorn G.W. (2014). LIPCAR: A mitochondrial lnc in the noncoding RNA chain?. Circ. Res..

[161] Clayton D.A. (2000). Transcription and replication of mitochondrial DNA. Hum. Reprod..

[162] Krasich R., Copeland W.C. (2017). DNA polymerases in the mitochondria: A critical review of the evidence. Front. Biosci..

[163] García-Gómez S., Reyes A., Martínez-Jiménez M.I., Chocrón E.S., Mourón S., Terrados G., Powell C., Salido E., Méndez J., Holt I.J. (2013). PrimPol, an archaic primase/polymerase operating in human cells. Mol. Cell.

[164] Rudd S.G., Bianchi J., Doherty A.J. (2014). PrimPol-A new polymerase on the block. Mol. Cell. Oncol..

[165] Sykora P., Kanno S., Akbari M., Kulikowicz T., Baptiste B.A., Leandro G.S., Lu H., Tian J., May A., Becker K.A. (2017). DNA Polymerase Beta Participates in Mitochondrial DNA Repair. Mol. Cell. Biol..

[166] Kaufman B.A., Van Houten B. (2017). POLB: A new role of DNA polymerase beta in mitochondrial base excision repair. DNA Repair.

[167] Singh B., Li X., Owens K.M., Vanniarajan A., Liang P., Singh K.K. (2015). Human REV3 DNA Polymerase Zeta Localizes to Mitochondria and Protects the Mitochondrial Genome. PLoS ONE.

[168] Wisnovsky S., Jean S.R., Kelley S.O. (2016). Mitochondrial DNA repair and replication proteins revealed by targeted chemical probes. Nat. Chem. Biol..

[169] Longley M.J., Ropp P.A., Lim S.E., Copeland W.C. (1998). Characterization of the native and recombinant catalytic subunit of human DNA polymerase gamma: Identification of residues critical for exonuclease activity and dideoxynucleotide sensitivity. Biochemistry.

[170] Spelbrink J.N., Toivonen J.M., Hakkaart G.A., Kurkela J.M., Cooper H.M., Lehtinen S.K., Lecrenier N., Back J.W., Speijer D., Foury F. (2000). In vivo functional analysis of the human mitochondrial DNA polymerase POLG expressed in cultured human cells. J. Biol. Chem..

[171] Chattopadhyay R., Wiederhold L., Szczesny B., Boldogh I., Hazra T.K., Izumi T., Mitra S. (2006). Identification and characterization of mitochondrial abasic (AP)-endonuclease in mammalian cells. Nucleic Acids Res..

[172] Akbari M., Keijzers G., Maynard S., Scheibye-Knudsen M., Desler C., Hickson I.D., Bohr V.A. (2014). Overexpression of DNA ligase III in mitochondria protects cells against oxidative stress and improves mitochondrial DNA base excision repair. DNA Repair.

[173] Barchiesi A., Wasilewski M., Chacinska A., Tell G., Vascotto C. (2015). Mitochondrial translocation of APE1 relies on the MIA pathway. Nucleic Acids Res..

[174] Szymanski M.R., Yu W., Gmyrek A.M., White M.A., Molineux I.J., Lee J.C., Yin Y.W. (2017). A domain in human EXOG converts apoptotic endonuclease to DNA-repair exonuclease. Nat. Commun..

[175] Sharma P., Sampath H. (2019). Mitochondrial DNA Integrity: Role in Health and Disease. Cells.

[176] Gammage P.A., Moraes C.T., Minczuk M. (2018). Mitochondrial Genome Engineering: The Revolution May Not Be CRISPR-Ized. Trends Genet..

[177] Nissanka N., Minczuk M., Moraes C.T. (2019). Mechanisms of Mitochondrial DNA Deletion Formation. Trends Genet..

[178] Srivastava S., Moraes C.T. (2001). Manipulating mitochondrial DNA heteroplasmy by a mitochondrially targeted restriction endonuclease. Hum. Mol. Genet..

[179] Srivastava S., Moraes C.T. (2005). Double-strand breaks of mouse muscle mtDNA promote large deletions similar to multiple mtDNA deletions in humans. Hum. Mol. Genet..

[180] Bacman S.R., Williams S.L., Garcia S., Moraes C.T. (2010). Organ-specific shifts in mtDNA heteroplasmy following systemic delivery of a mitochondria-targeted restriction endonuclease. Gene Ther..

[181] Minczuk M., Papworth M.A., Miller J.C., Murphy M.P., Klug A. (2008). Development of a single-chain, quasi-dimeric zinc-finger nuclease for the selective degradation of mutated human mitochondrial DNA. Nucleic Acids Res..

[182] Reddy P., Ocampo A., Suzuki K., Luo J., Bacman S.R., Williams S.L., Sugawara A., Okamura D., Tsunekawa Y., Wu J. (2015). Selective elimination of mitochondrial mutations in the germline by genome editing. Cell.

[183] Gammage P.A., Rorbach J., Vincent A.I., Rebar E.J., Minczuk M. (2014). Mitochondrially targeted ZFNs for selective degradation of pathogenic mitochondrial genomes bearing large-scale deletions or point mutations. EMBO Mol. Med..

[184] Gammage P.A., Gaude E., Van Haute L., Rebelo-Guiomar P., Jackson C.B., Rorbach J., Pekalski M.L., Robinson A.J., Charpentier M., Concordet J.-P. (2016). Near-complete elimination of mutant mtDNA by iterative or dynamic dose-controlled treatment with mtZFNs. Nucleic Acids Res..

[185] Bacman S.R., Williams S.L., Pinto M., Peralta S., Moraes C.T. (2013). Specific elimination of mutant mitochondrial genomes in patient-derived cells by mitoTALENs. Nat. Med..

[186] Gammage P.A., Viscomi C., Simard M.-L., Costa A.S.H., Gaude E., Powell C.A., Van Haute L., McCann B.J., Rebelo-Guiomar P., Cerutti R. (2018). Genome editing in mitochondria corrects a pathogenic mtDNA mutation in vivo. Nat. Med..

[187] Bacman S.R., Kauppila J.H.K., Pereira C.V., Nissanka N., Miranda M., Pinto M., Williams S.L., Larsson N.-G., Stewart J.B., Moraes C.T. (2018). MitoTALEN reduces mutant mtDNA load and restores tRNAAla levels in a mouse model of heteroplasmic mtDNA mutation. Nat. Med..

[188] Wang G., Shimada E., Zhang J., Hong J.S., Smith G.M., Teitell M.A., Koehler C.M. (2012). Correcting human mitochondrial mutations with targeted RNA import. Proc. Natl. Acad. Sci. USA.

[189] Chen H.-W., Rainey R.N., Balatoni C.E., Dawson D.W., Troke J.J., Wasiak S., Hong J.S., McBride H.M., Koehler C.M., Teitell M.A. (2006). Mammalian polynucleotide phosphorylase is an intermembrane space RNase that maintains mitochondrial homeostasis. Mol. Cell. Biol..

[190] Tarassov I., Entelis N., Martin R.P. (1995). An intact protein translocating machinery is required for mitochondrial import of a yeast cytoplasmic tRNA. J. Mol. Biol..

[191] Kolesnikova O.A., Entelis N.S., Jacquin-Becker C., Goltzene F., Chrzanowska-Lightowlers Z.M., Lightowlers R.N., Martin R.P., Tarassov I. (2004). Nuclear DNA-encoded tRNAs targeted into mitochondria can rescue a mitochondrial DNA mutation associated with the MERRF syndrome in cultured human cells. Hum. Mol. Genet..

[192] Comte C., Tonin Y., Heckel-Mager A.-M., Boucheham A., Smirnov A., Auré K., Lombès A., Martin R.P., Entelis N., Tarassov I. (2013). Mitochondrial targeting of recombinant RNAs modulates the level of a heteroplasmic mutation in human mitochondrial DNA associated with Kearns Sayre Syndrome. Nucleic Acids Res..

[193] Tonin Y., Heckel A.-M., Vysokikh M., Dovydenko I., Meschaninova M., Rötig A., Munnich A., Venyaminova A., Tarassov I., Entelis N. (2014). Modeling of antigenomic therapy of mitochondrial diseases by mitochondrially addressed RNA targeting a pathogenic point mutation in mitochondrial DNA. J. Biol. Chem..

[194] Schneider A., Martin J., Agabian N. (1994). A nuclear encoded tRNA of Trypanosoma brucei is imported into mitochondria. Mol. Cell. Biol..

[195] Kumar R., Maréchal-Drouard L., Akama K., Small I. (1996). Striking differences in mitochondrial tRNA import between different plant species. Mol. Gen. Genet..

[196] Kiss T., Filipowicz W. (1992). Evidence against a mitochondrial location of the 7-2/MRP RNA in mammalian cells. Cell.

[197] Jacobson M.R., Cao L.G., Wang Y.L., Pederson T. (1995). Dynamic localization of RNase MRP RNA in the nucleolus observed by fluorescent RNA cytochemistry in living cells. J. Cell Biol..

[198] Goldfarb K.C., Cech T.R. (2017). Targeted CRISPR disruption reveals a role for RNase MRP RNA in human preribosomal RNA processing. Genes Dev..

[199] Greber B.J., Boehringer D., Leitner A., Bieri P., Voigts-Hoffmann F., Erzberger J.P., Leibundgut M., Aebersold R., Ban N. (2014). Architecture of the large subunit of the mammalian mitochondrial ribosome. Nature.

[200] Brown A., Amunts A., Bai X.-C., Sugimoto Y., Edwards P.C., Murshudov G., Scheres S.H.W., Ramakrishnan V. (2014). Structure of the large ribosomal subunit from human mitochondria. Science.

[201] Greber B.J., Bieri P., Leibundgut M., Leitner A., Aebersold R., Boehringer D., Ban N. (2015). Ribosome. The complete structure of the 55S mammalian mitochondrial ribosome. Science.

[202] Lee Y.S., Shibata Y., Malhotra A., Dutta A. (2009). A novel class of small RNAs: tRNA-derived RNA fragments (tRFs). Genes Dev..

[203] Haussecker D., Huang Y., Lau A., Parameswaran P., Fire A.Z., Kay M.A. (2010). Human tRNA-derived small RNAs in the global regulation of RNA silencing. RNA.

[204] Ro S., Ma H.-Y., Park C., Ortogero N., Song R., Hennig G.W., Zheng H., Lin Y.-M., Moro L., Hsieh J.-T. (2013). The mitochondrial genome encodes abundant small noncoding RNAs. Cell Res..

[205] Geiger J., Dalgaard L.T. (2017). Interplay of mitochondrial metabolism and microRNAs. Cell. Mol. Life Sci..

[206] Bartel D.P. (2009). MicroRNAs: Target recognition and regulatory functions. Cell.

[207] Bandiera S., Rüberg S., Girard M., Cagnard N., Hanein S., Chrétien D., Munnich A., Lyonnet S., Henrion-Caude A. (2011). Nuclear outsourcing of RNA interference components to human mitochondria. PLoS ONE.

[208] Barrey E., Saint-Auret G., Bonnamy B., Damas D., Boyer O., Gidrol X. (2011). Pre-microRNA and mature microRNA in human mitochondria. PLoS ONE.

[209] Sripada L., Tomar D., Prajapati P., Singh R., Singh A.K., Singh R. (2012). Systematic analysis of small RNAs associated with human mitochondria by deep sequencing: Detailed analysis of mitochondrial associated miRNA. PLoS ONE.

[210] Jagannathan R., Thapa D., Nichols C.E., Shepherd D.L., Stricker J.C., Croston T.L., Baseler W.A., Lewis S.E., Martinez I., Hollander J.M. (2015). Translational Regulation of the Mitochondrial Genome Following Redistribution of Mitochondrial MicroRNA in the Diabetic Heart. Circ. Cardiovasc. Genet..

[211] Hashimoto Y., Ito Y., Niikura T., Shao Z., Hata M., Oyama F., Nishimoto I. (2001). Mechanisms of neuroprotection by a novel rescue factor humanin from Swedish mutant amyloid precursor protein. Biochem. Biophys. Res. Commun..

[212] Hashimoto Y., Niikura T., Tajima H., Yasukawa T., Sudo H., Ito Y., Kita Y., Kawasumi M., Kouyama K., Doyu M. (2001). A rescue factor abolishing neuronal cell death by a wide spectrum of familial Alzheimer’s disease genes and Abeta. Proc. Natl. Acad. Sci. USA.

[213] Lee C., Yen K., Cohen P. (2013). Humanin: A harbinger of mitochondrial-derived peptides?. Trends Endocrinol. Metab..

[214] Lee C., Zeng J., Drew B.G., Sallam T., Martin-Montalvo A., Wan J., Kim S.-J., Mehta H., Hevener A.L., de Cabo R. (2015). The mitochondrial-derived peptide MOTS-c promotes metabolic homeostasis and reduces obesity and insulin resistance. Cell Metab..

[215] Cobb L.J., Lee C., Xiao J., Yen K., Wong R.G., Nakamura H.K., Mehta H.H., Gao Q., Ashur C., Huffman D.M. (2016). Naturally occurring mitochondrial-derived peptides are age-dependent regulators of apoptosis, insulin sensitivity, and inflammatory markers. Aging.

[216] Kim S.-J., Xiao J., Wan J., Cohen P., Yen K. (2017). Mitochondrially derived peptides as novel regulators of metabolism. J. Physiol..

